# New record of *Miniopterusmagnater* (Chiroptera, Miniopteridae) from south-western China and a comparative study of three species of *Miniopterus* in China

**DOI:** 10.3897/BDJ.12.e129879

**Published:** 2024-09-13

**Authors:** Yishun Qian, Xin Mou, Wen Wang, Wenxiang Zhang, Yuanyuan Li, Li Wu, Canjun Zhao, Zhiwei Jiao, Song Li

**Affiliations:** 1 Kunming Natural History Museum of Zoology, Kunming Institute of Zoology, Chinese Academy of Sciences, Kunming, China Kunming Natural History Museum of Zoology, Kunming Institute of Zoology, Chinese Academy of Sciences Kunming China; 2 Huanglianshan National Nature Reserve in Yunnan Province, Honghe Hani and Yi Autonomous, China Huanglianshan National Nature Reserve in Yunnan Province Honghe Hani and Yi Autonomous China; 3 Yunnan Academy of Forestry and Grassland, Kunming, China Yunnan Academy of Forestry and Grassland Kunming China; 4 Yunnan Key Laboratory of Biodiversity of Gaoligong Mountain, Kunming, China Yunnan Key Laboratory of Biodiversity of Gaoligong Mountain Kunming China; 5 Gaoligong Mountain Forest Ecosystem Observation and Research Station of Yunnan Province, Kunming, China Gaoligong Mountain Forest Ecosystem Observation and Research Station of Yunnan Province Kunming China; 6 Cangshan Erhai National Nature Reserve Administration, Dali Bai Autonomous Prefecture, China Cangshan Erhai National Nature Reserve Administration Dali Bai Autonomous Prefecture China; 7 Yunnan Key Laboratory of Biodiversity Information, Kunming Institute of Zoology, Chinese Academy of Sciences, Kunming, China Yunnan Key Laboratory of Biodiversity Information, Kunming Institute of Zoology, Chinese Academy of Sciences Kunming China

**Keywords:** new record, *Miniopterusmagnater*, morphology, south-western China

## Abstract

**Background:**

This research documents a new record of *Miniopterusmagnater* in the south-western region of China, a significant discovery given the limited diversity of the *Miniopterus* genus within the country. Only three species of *Miniopterus* occur in China: *Miniopterusmagnater*, *Miniopterusfuliginosus* and *Miniopteruspusillus*. These species share a high degree of morphological similarity, particularly in their external characteristics. This underscores the necessity for the identification of additional distinguishing traits that can aid in the taxonomic differentiation of these closely-related species.

**New information:**

During the 2023 field expedition to various nature reserves in Yunnan Province, China, we encountered specimens of the genus *Miniopterus*. Utilising a combination of morphological assessments and phylogenetic analyses, we identified six individuals as *Miniopterusmagnater*. A review of the existing geographical distribution data revealed that this species is primarily found in central and southern regions of China, with no previous records from the south-western part of the country. Based on our findings, we present a novel record of *Miniopterusmagnater*'s distribution in the south-western region of China.

## Introduction

Although Miniopteridae used to be a subfamily of Vespertilionidae, it was later found that miniopterines differed from vespertilionids in numerous aspects of morphology and genetics in many ways (Agrawal and Sinha 1973, Mein and Tupinier 1977, Krutzsch and Crichton 1990, Reep and Bhatnagar 2000, Kawai et al. 2002, Hoofer and Bussche 2003, Hutcheon and Kirsch 2004, Van Den Bussche and Hoofer 2004, Eick et al. 2005, Miller-Butterworth et al. 2007). Thus, the taxonomic status of Miniopteridae was elevated to the family level. The family Miniopteridae now stands as a distinct entity, encompassing the monotypic genus *Miniopterus*. This genus is distinguished by a pronounced elongation of the third finger and second phalanx, a unique morphological trait amongst its bat counterparts. Despite a high degree of similarity in external morphology amongst the species within this genus, the primary taxonomic differentiators lie in the size and proportional length of their wings. Historically, *Miniopterus* species identification and classification relied predominantly on fur colour and other morphological markers; however, recent advances in molecular systematics and biogeographical research have unveiled a complex picture of *Miniopterus* species taxonomy, revealing a degree of previous misclassification (Appleton et al. 2004) . Notably, the taxonomic delineation of *Miniopterusschreibersii* has been a subject of particular scrutiny, with ongoing research aiming to resolve their classification (Miller-Butterworth et al. 2007).

Historically, numerous species of the *Miniopterus* genus in the Asian Region were broadly categorised as *Miniopterusschreibersii* Kuhl, 1817 (Wilson and Reeder 2005). However, with the advancement of molecular biology and the progression of scientific research, a multitude of new species have been progressively delineated and identified. The prevailing taxonomic consensus in China posits that the taxon previously referred to as *Miniopterusschreibersii* in China should be reclassified as *Miniopterusfuliginosus*. This reclassification is supported by a synthesis of evidence encompassing morphological traits, genetic data and biogeographical distribution (Miller-Butterworth et al. 2007, Li et al. 2015). Many species within this genus exhibit overlapping characteristics, particularly in body size and fur colouration. However, there are also studies that rely on these external features to distinguish between species (Skinner and Chimimba 2005).

*Miniopterusfuliginosus* was formerly in taxonomic confusion with *Miniopterusschreibersii* (Li et al. 2015). Nonetheless, genetic analyses have uncovered substantial genetic divergence at the DNA level between these two taxa, providing robust support for their classification as distinct species (Miller-Butterworth et al. 2007). Furthermore, *Miniopterusschreibersii*, a species characteristic of the Mediterranean Region, is predominantly found across Europe and western Asia, whereas *Miniopterusfuliginosus* is more prevalent in China and its neighbouring regions (Piksa and Gubała 2020). In China, the genus *Miniopterus* encompasses three species: *Miniopterusfuliginosus*, *Miniopterusmagnater* and *Miniopteruspusillus*. Despite extensive morphological overlap between *Miniopterusmagnater* and *Miniopterusfuliginosus*, discernible differences emerge in forearm length and cranial dimensions. A critical taxonomic criterion is the width of the upper jaw's third molar, with *Miniopterusmagnater* exceeding 7.3 mm and *Miniopterusfuliginosus* measuring less than this threshold (Smith and Xie 2008).

During field investigations in key natural reserves of Yunnan Province, China, including Cangshan Erhai National Nature Reserve, Huanglianshan National Nature Reserve and Guanyinshan Provincial Nature Reserve, we collected specimens of the three Chinese *Miniopterus* species. Our geographical distribution analysis revealed that *Miniopterusmagnater* is predominantly found in southern Asia and Southeast Asia, as documented by Han et al. (2008) and Francis et al. (2010). This species has been recorded in southern regions such as Hainan, Guangdong and Hong Kong (Smith and Xie 2008), extending to Fujian and Anhui in eastern China (Han et al. 2008). However, until now, no distribution records existed for *Miniopterusmagnater* in south-western China.

## Materials and methods

### Sample collection

**Figure 1.**
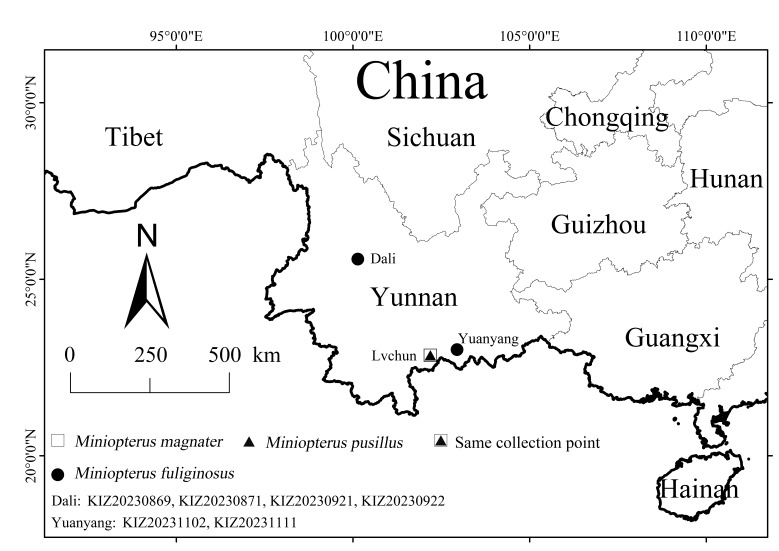
The collection sites of the specimens used in this study.

The specimens used in this study were collected using mist-nets in 2023 during field expeditions to three nature reserves in Yunnan, China (Fig. 1). *M.magnater* and *M.pusillus* were collected in April 2023 from the Huanglianshan National Nature Reserve, Luchun County, Yunnan, China (N22.85°, E102.19°, altitude 866 m). *M.fuliginosus* was collected from two different localities: the Cangshan Erhai National Nature Reserve in Dali Bai Autonomous Prefecture, Yunnan, China (25.57 °N, 100.14 °E, altitude 1912 m, September 2023) and Yuanyang Guanyinshan Provincial Nature Reserve of Yunnan, China (N23.01 °, E102.94 °, altitude 2401 m, October 2023) (Bates and Harrison 1997). Specimens were preserved by immersion in absolute ethanol. The specimens were deposited at the Kunming Natural History Museum of Zoology, Kunming Institute of Zoology, Chinese Academy of Sciences (KIZ, CAS). *M.magnater* collection numbers are KIZ20230213, KIZ20230270, KIZ20230274, KIZ20230276, KIZ20230307 and KIZ20230312. The specimen collection numbers of *M.fuliginosus* are KIZ20230869, KIZ20230871, KIZ20230921, KIZ20230922, KIZ20231102 and KIZ20231111. The collection numbers of *M.pusillus* are KIZ20230263, KIZ20230265, KIZ20230305, KIZ20230306, KIZ20230311 and KIZ20230314.

### Morphological description and measurement

Five external morphologies: tail length (TL), head-body length (HB), forearm length (FA), tibia length (TIB) and ear length (EAR) were measured in the field (Bates and Harrison 1997). The skull specimen was stripped under experimental conditions and the attached muscle tissue on the skull was carefully removed to maintain the integrity of the skull structure. A digital vernier caliper was used to measure the skull, the measurement accuracy being 0.01 mm. In terms of skull morphology measurement, we selected seven features for measurement: greatest skull length, from the anterior rim of the alveolus of the first upper incisor to the most projecting point of the occipital region (GSKL); postorbital breadth, narrowest dorsal width posterior to the postorbital constriction of the cranium (POB); mastoid width, the greatest width across the mastoid region (MAW); mandible length, from the posterior-most point of the condyle to anterior-most alveoli of lower incisors (ML); width across upper canines, taken across the outer-most points of the crowns of the upper canines (C-C); width across the upper third molars, taken across the outer-most point of the crowns of the 3^rd^ upper molars (M3-M3); complete upper canine-molar tooth row, taken from the anterior-most point of the crown of the upper canine to the posterior-most point of the crown of 3^rd^ upper molar (C-M3). Skull measurements were based on bat studies of the genus *Pteropus* in India and Sri Lanka (Kusuminda et al. 2022). The measurements of bat wing bones are undertaken following Bates and Harrison (1997); mt: metacarpal, from the extremity of the carpus to the distal extremity of the metacarpal. Ph: phalanx of the metacarpal, taken from the proximal to the distal extremity of the phalanx. For example: 1ph3mt, refers to the first phalanx of the third metacarpal. 2ph3mt/4mt/1ph4mt/5mt/1ph5mt: as above. It is worth noting that we did not take in situ measurements of the hand skeleton of *M.pusillus* during the field survey. Therefore, the morphological characteristics of the wings of *M.pusillus* were measured by using specimens immersed in absolute ethanol. Since the morphology of the hand bones did not change, most of the characteristics were considered to be reliable and not much different from those when they were alive in the natural state. During the assessment, it was observed that the second phalanx of the wing exhibited significant curling, a condition not conducive to accurate measurement. Consequently, these specimens had to be manipulated into a straight configuration for measurement, an intervention that may introduce discrepancies between the measured data and the specimens' natural state. Due to these methodological limitations, the morphological data derived from the second phalanx were deemed unreliable for inclusion in our analysis. These measurements were conducted by a single individual using consistent equipment, thereby minimising the influence of human error on the data.

### Data processing

Data processing was undertaken using the IBM SPSS Statistics v. 27.0.1 non-parametric 1-sample K-S (Kolmogorov-Smirnov) test (Berger and Zhou 2014). Inspection of the morphological data obtained by our measurements showed that all morphological data conformed to a normal distribution. Analysis of variance (ANOVA) was used to determine whether there were significant differences in morphological parameters amongst species and principal component analysis (PCA) was performed on the morphological measurements of specimens.

### Phylogenetic analysis

Genomic DNA from bat muscle tissue preserved in anhydrous ethanol was extracted using the TSP202-200 Animal Genomic DNA Extraction Kit (Bejing Tsingke Biotech Co., Ltd., Chian). We used the primers Molcit-F (5'-AATGACATGAAAAATCACCGTTGT3', Ibáñez et al. (2006)) and Cytb-H (5'-CTTTTCTGGTTTACAAGACCAG-3', Weyeneth et al. (2008)) to amplify the complete mitochondrial cytochrome b (cyt-b) gene. Polymerase chain reaction (PCR) was performed in a 50 μl system. The system contained 45 μl of enzyme: Gold Mix (green), 2 μl of forward primer, 2 μl of reverse primer and 1 μl of DNA template. The PCR protocol required an initial denaturation at 94°C for 2 minutes, followed by five cycles of denaturation at 94°C for 30 seconds, annealing at 50°C for 30 seconds and extension at 72°C for 1 minute, followed by 35 additional cycles: denaturation at 94°C for 30 s, annealing at 55°C for 40 s, extension at 72°C for 1 min and final extension at 72°C for 10 min and renaturation at 4°C. Finally. purifed samples were sequenced by the ABI 3730XL DNA Analyzer (USA) at Beijing Tsingke Biotech Co., Ltd (China).

The evolutionary history was inferred by using the Maximum Likelihood method and Hasegawa-Kishino-Yano model (Hasegawa et al. 1985), which has the highest log likelihood (-7525.55). The percentage of trees in which the associated taxa clustered together is shown next to the branches. Initial tree(s) for the heuristic search were obtained automatically by applying Neighbour-Joining and BioNJ algorithms to a matrix of pairwise distances estimated using the Maximum Composite Likelihood (MCL) approach and then selecting the topology with superior log likelihood value. A discrete Gamma distribution was used to model evolutionary rate differences amongst sites (5 categories (+*G*, parameter = 0.4761)). The rate variation model allowed for some sites to be evolutionarily invariable ([+*I*], 30.02% sites). This analysis involved 43 nucleotide sequences. There were a total of 1141 positions in the final dataset. Evolutionary analyses were conducted in MEGA11 (Tamura et al. 2021). We selected Cyt b sequences provided by other researchers from the National Center for Biotechnology Information (NCBI) database for comparative study. Amongst them, *Kerivoulafurva* was used as the outgroup. The relevant situation is shown in the following table (Table 1).

The number of base substitutions per site from between sequences are shown (Suppl. material 1). Analyses were conducted using the Maximum Composite Likelihood model (Tamura et al. 2004). This analysis involved 21 nucleotide sequences. Codon positions included were 1st+2nd+3rd+Noncoding. All ambiguous positions were removed for each sequence pair (pairwise deletion option). There were a total of 1141 positions in the final dataset. Evolutionary analyses were conducted in MEGA11 (Tamura et al. 2021). The relevant sequences of *M.magnater*, *M.fuliginosus* and *M.pusillus* used are detailed in Table 1.

## Taxon treatments

### Miniopterus magnater

Sanborn, 1931

9ADB5014-35F8-5D8F-8E8E-2DD7EA235EF3

https://www.gbif.org/species/2432503

#### Materials

- **Type status:** Other material. **Occurrence:** occurrenceID: A45CA4E8-576D-52FD-A7F4-A17A0FDE9E7E; **Taxon:** kingdom: Animalia; phylum: Chordata; class: Mammalia; order: Chiroptera; family: Miniopteridae; genus: Miniopterus; **Location:** countryCode: China; stateProvince: Yunnan; county: Lvchun; locality: Huanglianshan National Nature Reserve; verbatimCoordinates: N22.85°, E102.19°

#### Diagnosis

Dorsal pileus long, soft, light brownish-black; abdominal pileus dark brown, tips of pileus lighter in colour. Large body size. Head-body length: 58-75 mm, tail length: 52-64 mm, hind-foot length: 9-13 mm, ear length: 11-17 mm, forearm length: 47-54 mm. Total length of skull more than 17 mm. Condylobasal length more than 14 mm, width across the upper third molars more than 7.4 mm (Smith and Xie 2008).

### Miniopterus fuliginosus

Hodgson, 1835

D276007B-E3D8-5446-A71D-1508B6B729AA

https://www.gbif.org/zh/species/5787699

#### Materials

- **Type status:** Other material. **Occurrence:** occurrenceID: 11EC16D1-79EE-5658-8B51-21819D99F77B; **Taxon:** kingdom: Animalia; phylum: Chordata; class: Mammalia; order: Chiroptera; family: Miniopteridae; genus: Miniopterus; **Location:** country: China; stateProvince: Yunnan

#### Diagnosis

Wholly sooty brown. Ears, lips and muzzle, as in the last: and face sharp, but the rostrum somewhat recurved, owing to the concave bend of the nasal bones, which in formosa are rather convex (Hodgson 1835). Transverse width of maxillary third molars less than or equal to 7.3 mm (Smith and Xie 2008).

### Miniopterus pusillus

Dobson, 1876

B3804FEC-1FD2-5BA5-AC07-1EE8AEBCF04A

https://www.gbif.org/species/2432506

#### Materials

- **Type status:** Other material. **Occurrence:** occurrenceID: 91B5C895-7477-5C93-BA6F-50AC0F35F3FC; **Taxon:** kingdom: Animalia; phylum: Chordata; class: Mammalia; order: Chiroptera; family: Miniopteridae; genus: Miniopterus; **Location:** country: China; county: Yunnan

#### Diagnosis

The fur is dark brown in colour, with some extension of the fur on to the tail. Head-body length 45-48 mm, tail length: 40-48 mm, hind-foot length: 7-8 mm, ear length 10-11 mm, forearm length 39-42 mm, total cranial length 13.5-14.5 mm. The skull has no sagittal crest (Smith and Xie 2008).

## Analysis

### Morphological characteristics

**Body and Fur**

**Figure 2.**
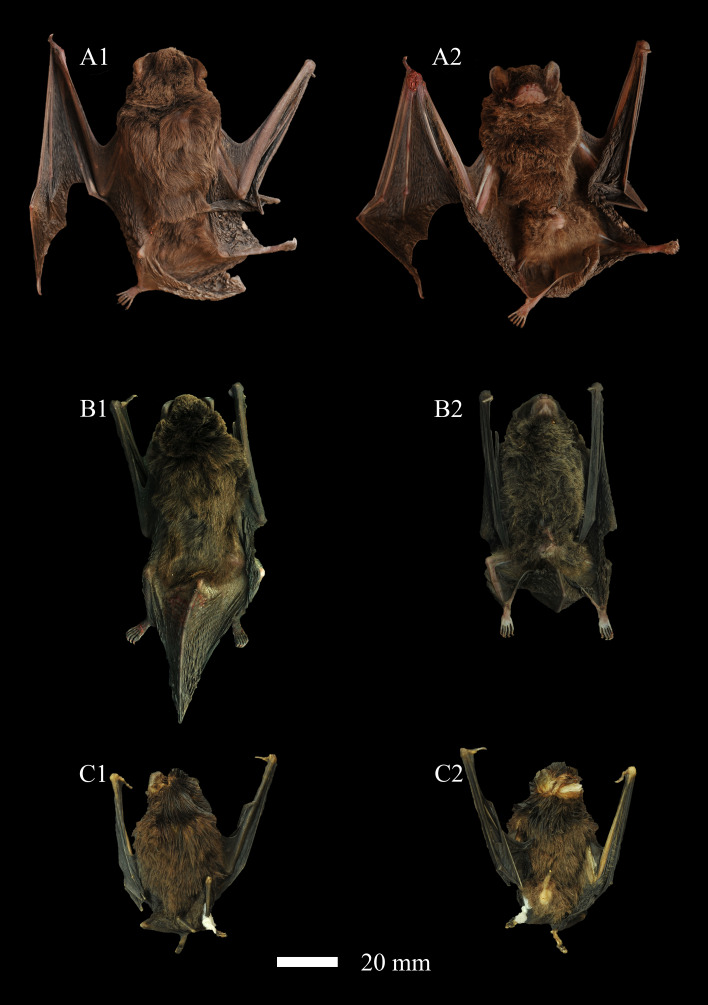
Three specimens of *Miniopterus* used in this study. **A** *Miniopterusmagnater*, specimen collection number: KIZ20230213; **B** *Miniopterusfuliginosus*, specimen collection number: KIZ20231111; **C** *Miniopteruspusillus*, specimen collection number: KIZ20230306, specimens preserved in anhydrous ethanol. 1 Dorsal view; 2 Ventral view.

*M.magnater* is characterised by a slender body plan, featuring an oval head, rounded ears with distinct tragus and a well-developed thumb. The second phalanx of the third metacarpal (2ph3mt) is notably elongated, approaching approximately 90% of the forearm length (FA). The FA is robust and covered with dense fur on the ventral aspect, while the mouth area is devoid of villi. The hind limbs are well-developed, presenting five distinct toes and the genitalia are well-developed. The body of *M.magnater* is densely covered with downy hair, presenting an overall tan hue, while the villi tips are marginally golden brown. The colouration along individual villi remains largely uniform from the tail root to the head, without a pronounced gradient. Both the dorsal and ventral body surfaces, as well as the head to tail hair colouration, exhibit consistency, with no significant variation (Fig. 2).

**Skull**

**Figure 3.**
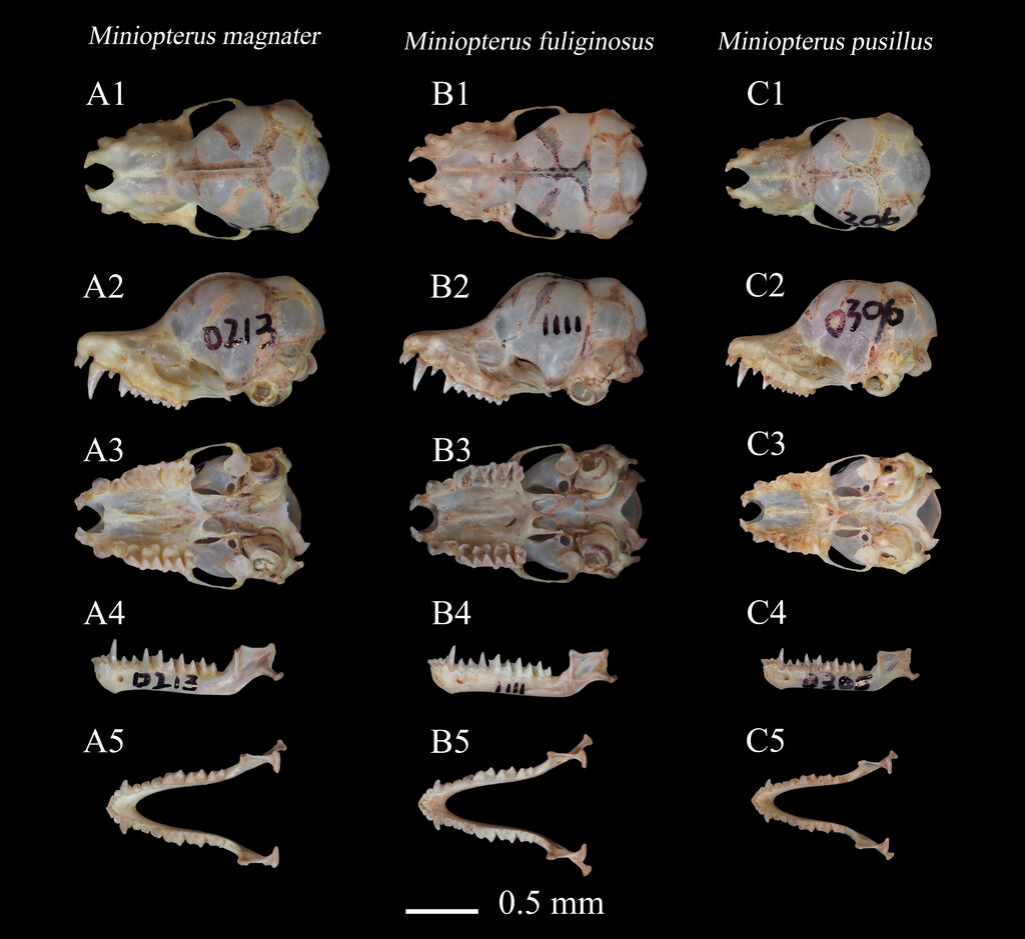
Photograph of the skulls, mandibles, specimens. **A** *Miniopterusmagnater*, collection number: KIZ20230213; **B** *Miniopterusfuliginosus*, collection number: KIZ20231111; **C** *Miniopteruspusillus*, collection number: KIZ20230306. 1 dorsal view of the cranium; 2 lateral view of skull; 3 ventral view of skull; 4 lateral view of the mandible; 5 occlusal view of mandible.

In the dorsal view, the anterior maxilla presents a concave oval configuration, with the incisors exhibiting a distinct inward retraction. The sagittal suture is prominently defined. The canines are markedly prominent and the orbits are notably large. The zygomatic arches are characterised by a straight lateral profile, with robust anterior and posterior extremities and a more delicate mid-section. From a lateral perspective, the skull of *M.magnater* is elegantly slender, featuring a subtle concavity at the maxillary level and a pronounced sagittal suture. The occipital region of the skull is broadly oval, with a significant indentation observable on the parietal bones. The zygomatic processes are well-developed, with the anterior portion being broad and the posterior tapering to a narrower point. Relative to *M.fuliginosus* and *M.pusillus*, the skull of *M.magnater* is notably elongated and the sagittal suture is comparatively more developed (Fig. 3).

**Dentition**

Dental formula of the *M.magnater* : 2.1.2.3/3.1.3.3 = 36. The base of the canines of the cranium protrudes outwards and the first premolar is depressed medially, canine developed. First premolar is small, not as high as the incisor, the height of first premolar is not half that of second premolar, second premolar is higher in height than the posterior teeth, the height of the molar almost the same. Well-developed canines in the lower jaw, the height of first premolar and second premolar is basically the same and third premolar is significantly higher than first premolar and second premolar, the height of the molars decreases sequentially from front to back (Fig. 3).

**Morphological measurement data**

The descriptive statistics, as delineated in Table 2, reveals pronounced distinctions between *M.magnater*, *M.fuliginosus* and *M.pusillus*. Amongst the trio of species evaluated, *M.magnater* emerges as the most substantial in stature. Owing to the morphological congruence observed on external examination, we measured some morphological data of the skull and wings. The cranial measurements for *M.magnater* align well with prior scholarly works (Hodgson 1835, Smith and Xie 2008). Variability is noted in the extremities of the measured range; however, the mean values, juxtaposed with the standard deviation of these metrics, indicate a higher degree of precision in the cranial measurements and suggest limited inter-individual variability within *M.magnater*.

By means of morphometric data, the specimens of the three species of the genus Miniopterus collected in this study are in general agreement with the morphometric data of other scholars (Hodgson 1835, Smith and Xie 2008, Saikia et al. 2020, Kusuminda et al. 2022). Thus, we have reason to believe that these specimens belong to three species of Miniopterus, *M.magnater* , *M.fuliginosus* and *M.pusillus*.

### Comparison of significant differences

By analysis of skulls based on measurement data, *M.magnater* demonstrates significant morphological distinctions from both *M.fuliginosus* and *M.pusillus*, as evidenced by pronounced differences in GTL, POB, MAW, M3-M3, C-M3, C-C and ML, with statistical significance indicated by a P-value of less than 0.01. In the context of the wing skeleton, 3mt, 4mt and 5mt of *M.magnater* and *M.fuliginosus* do not exhibit significant variability (P > 0.05, with a minimum P-value of 0.257). However, 1ph3m and 1ph4mt display highly significant differences. 1ph5mt presents significant to extremely significant differences, as detailed in Table 3 and Suppl. material 2.

By performing principal component analysis on the morphometric data, we obtained three principal components. The eigenvalue of principal component 1 is 15.894 and Variance explained is 88.048%. The eigenvalue and Variance showed that the differences between component 2 and component 3 were relatively small and the cumulative variance contribution was 95.815%. Except for TL, E, 3mt, 4mt, 5mt and ML, which have relatively low loadings, the other eigen loadings are relatively high. Overall, there was less loss of original information, which was suitable for principal component analysis (Table 4).

**Figure 4.**
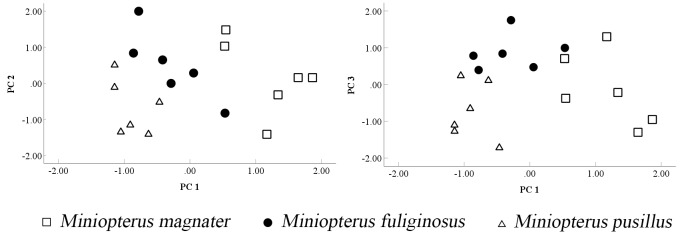
Scatterplots of the samples for principal component factors 1 vs. 2 and 1 vs. 3, respectively.

The scatter plots, generated from the principal component analysis, distinctly delineate the three Miniopterus species, with no overlap observed between *M.magnater*, *M.fuliginosus* and *M.pusillus*. The data points exhibit a clear demarcation, forming three discrete groups (Fig. 4).

### Phylogenetic analysis

**Figure 5.**
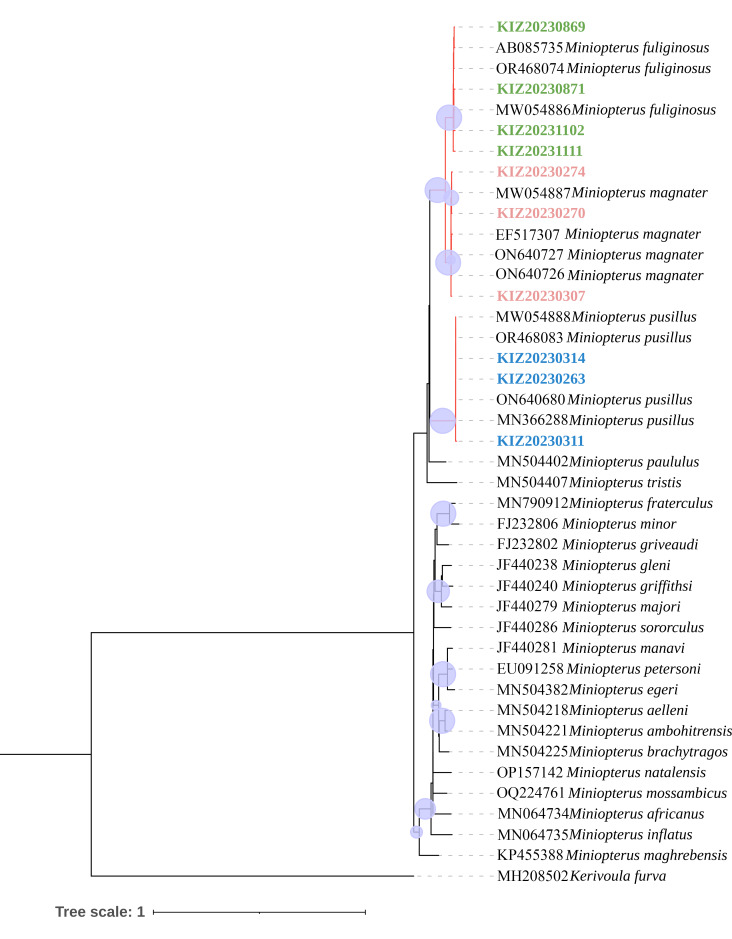
Phylogenetic tree of the genus *Miniopterus*, based on the cytb gene constructed using the Maximum Likelihood method, nodes with posterior probabilities < 0.70 are not labelled. The percentage of trees in which the associated taxa clustered together is shown next to the branches，represented by blue circles, with larger graphs indicating higher values.

Phylogenetic analyses have confirmed that our cytochrome b (Cyt b) sequences, in conjunction with those from other researchers, form a monophyletic group with *M.fuliginosus*, *M.magnater* and *M.pusillus*. The sequences display no evidence of hybrid relationships amongst the three species. These phylogenetic outcomes are congruent with the findings from morphological studies, thereby reinforcing the validity of our species identifications (Fig. 5).

Using the genetic distance table, we can see that, within the populations of all three species, the differences in genetic distances between the sequences of the specimens collected in this study and those of other researchers are relatively small, with an average difference of approximately 0.75% for *M.magnater*, 1.09% for *M.fuliginosus* and 0.20% for *M.pusillus* . In terms of inter-population variation, the difference in genetic distance between *M.magnater* and *M.fuliginosus* was relatively small, with a mean difference of about 6.39%. The mean difference in genetic distance between *M.magnater* and *M.pusillus* was about 13.40%. The mean difference in genetic distance between *M.fuliginosus* and *M.pusillus* is about 14.02% (Suppl. material 1). The results of genetic distances indicate that the specimens we collected in Yunnan Province, China, are indeed *M.magnater* , *M.fuliginosus* and *M.pusillus* belonging to the genus *Miniopterus*.

### Comparison of differences

Comparative analysis of coat colouration reveals marked differences on the body surface between *M.magnater* and its congeners, *M.fuliginosus* and *M.pusillus*. The latter two species display a predominantly grey-black coat, with more pronounced golden hues at the fur tips.

Comparative morphological assessments reveal significant differences in overall morphology and size between *M.magnater* and its congeners, *M.fuliginosus* and *M.pusillus*, while *M.magnater* and *M.fuliginosus* exhibit relatively minor variations in body size. Forearm length, body length and hind foot length are markedly longer in *M.magnater* than in *M.fuliginosus*. In contrast, there is a substantial disparity in body size when comparing *M.magnater* with the smaller *M.pusillus*.

Morphometric data analysis indicates significant differences in the mean values of tail length (TL), forearm length (FA), head-body length (HB), tibia length (TIB) and ear length (E) between populations of *M.magnater* and *M.fuliginosus* (Table 2). Intraspecific body size variations within *M.magnater* are also evident, as demonstrated by the standard deviation of the morphometric measurements.

There was no significant difference in the arrangement structure of the teeth between *M.magnater* and *M.fuliginosus* and *M.pusillus*. According to previous studies, the M3-M3 of *M.magnater* was greater than 7.3 mm, M3-M3 of M.fuliginosus less than 7.3 mm (Smith and Xie 2008) and all specimens of *M.magnater* and *M.fuliginosus* in this study conformed to this characteristic (Fig. 3).

In terms of skull differences, of the three species, the smallest skull of *M.pusillus* differed considerably from the other two, with no sagittal suture present in the skull. The size difference between *M.magnater* and *M.fuliginosus* was relatively small, with both having a distinct sagittal suture.

## Discussion

There are several Miniopterus species in the neighbouring countries of China that are more similar to the *Miniopterus* species distributed in China mentioned in this study. The new species *M.srinii* discovered in 2023 is very similar to *M.pusillus*, with forearm lengths ranging from 38.93 mm to 41.29 mm. The fur is dark golden yellow to dark brown and lighter than *M.pusilus* (Srinivasulu and Srinivasulu 2023). The newly-discovered *M.phillipsi* (2022) is somewhat smaller in external body size and cranial dimensions than *M.magnater* and *M.fuliginosus*, but is larger than *M.pusillus*. Its morphometric data overlap slightly with *M.fuliginosus* (Kusuminda et al. 2022). These four species are also similar in appearance, making it more difficult to distinguish them under field conditions. The distribution of *M.eschscholtzii* in the Philippines, which was previously recognised as a subspecies of *M.schreibersii* (Wilson and Mittermeier 2019), was made independent as a separate valid species by genetic studies of it (Miller-Butterworth et al. 2007), much like the change in taxonomic status of *M.fuliginosus*.

In the initial phase of mandibular morphological identification, a distinct diastema at the base of the teeth between the first (p1) and second (p2) premolars was observed in *M.fuliginosus*, a characteristic not present in *M.magnater*. At first, we considered this feature as a potential morphological factor in distinguishing the two species. However, with an increasing sample size, the consistency of this gap as a distinguishing feature proved to be variable; it was present in some individuals, but absent in others. The utility of such variations as indicators of ecological environments warrants further investigation and refinement. The observed overlap in body size metrics between *M.magnater* and *Mi.fuliginosus* underscores the challenge of species identification, based solely on external morphological characteristics.

Li et al. (2015) conducted a comparative study on *Miniopterusmagnater* and *Miniopterusfuliginosus* populations in Vietnam and China, revealing that the M3-M3 measurement in all individuals was less than 7.3 mm, with *M.magnater* specifically ranging from 7.03 mm to 7.29 mm and *Miniopterusfuliginosus* from 6.33 mm to 7.16 mm. Subsequent research by Saikia et al. (2020) and Kusuminda et al. (2022) reported M3-M3 measurements within a narrower range of 7.0 to 7.7 mm. In contrast, the present study observed measurements ranging from 7.30 to 7.60 mm. These findings suggest that the diagnostic feature of an M3-M3 width exceeding 7.3 mm may not be entirely reliable, as the majority of individuals exhibited M3-M3 widths greater than this threshold, albeit with some smaller individuals displaying measurements below it. Moreover, discrepancies in other morphometric measurements have been noted, with size overlap occurring between smaller *M.magnater* and larger *M.fuliginosus* individuals, complicating morphological identification.

In terms of genetic distance (Suppl. material 1), the genetic distance between *M.magnater* and *M.fuliginosus* is relatively close, only about 6%. Additionally, the genetic distance between *M.pusillus* with *M.magnater* and *M.fuliginosus* is relatively far higher than 13%. This result coincides with the differences in the morphometric data, where the similarity between *M.magnater* and *M.fuliginosus* is higher, the similarity of *M.pusillus* with *M.magnater* and *M.fuliginosus* was relatively low. As can be seen through the phylogenetic tree, these three species being on the same branch (Fig. 5). Although there are some significant differences between their three species, this may explain why similarities exist amongst the three species.

In Asia, *M.magnater* exhibits a broad distribution across the Indochina Peninsula, whereas *M.fuliginosus* is sparsely found in the same region, but is present in central and northern China (Li et al. 2015,Kusuminda et al. 2022). The distribution patterns of these species appear to be influenced by the complex topography of southern China, with the south-western distribution limited by the Hengduan Mountains and the Yunnan-Guizhou Plateau, the southern range constrained by the Nanling Mountains and the central region obstructed by the Wuyi Mountains. These geographical features impede their inland penetration, allowing only a few individuals to overcome such barriers. It is plausible that the prolonged geographical isolation has led to divergent evolutionary trajectories, adapting to their respective niches.

In southwest China, similar altitudes and environments, which are numerous, are being increasingly impacted by urbanisation, agricultural and forestry development and intensified human activities, leading to a reduction in suitable bat habitats. The establishment of nature reserves undoubtedly provides protection for bats and facilitates their survival and reproduction. Increasing the publicity of science and enhancing the construction and management of nature reserves are of positive significance for bats, wild animals, the protection of natural ecological environment and the sustainable development of human civilisation.

## Supplementary Material

XML Treatment for Miniopterus magnater

XML Treatment for Miniopterus fuliginosus

XML Treatment for Miniopterus pusillus

5BA1CB03-F7F6-5360-873A-2B9EAF9F5B1D 10.3897/BDJ.12.e129879.suppl1 Supplementary material 1 Uncorrected P-distances (%) amongst sequences. Data type Table Brief description Table of genetic distances of three species of the genus *Miniopterus* distributed in China. File: oo_1114285.docx https://binary.pensoft.net/file/1114285 Yishun Qian

CD4BD007-849A-5600-970D-E45F8ED0C679 10.3897/BDJ.12.e129879.suppl2 Supplementary material 2 LSD multiple comparisons Data type Table Brief description Results of LSD multiple comparisons of morphometric data of three specimens of species of the genus *Miniopterus* collected in this study. File: oo_1114792.docx https://binary.pensoft.net/file/1114792 Yishun Qian

## Figures and Tables

**Table 1.** The GenBank accession numbers and specimen collection numbers of the cyt b sequences used in the construction of the phylogenetic tree.

Species	GenBank accession numbers or specimen collection numbers	Reference	Species	GenBank accession numbers	Reference
*M.magnater*	KIZ20230314	This study	*M.fraterculus*	MN790912	Moir et al. (2020)
*M.magnater*	KIZ20230311	This study	*M.tristis*	MN504407	Demos et al. (2019)
*M.magnater*	KIZ20230263	This study	*M.inflatus*	MN064735	Lutz et al. (2019)
*M.magnater*	MW054887	Ruedi et al. (2021)	*M.minor*	FJ232806	Weyeneth et al. (2008)
*M.magnater*	ON640726	Wu et al. (2022)	*M.manavi*	JF440281	Ramasindrazana et al. (2011)
*M.magnater*	ON640727	Wu et al. (2022)	*M.majori*	JF440279	Ramasindrazana et al. (2011)
*M.magnater*	EF517307	Cui et al. (2007)	*M.gleni*	JF440238	Ramasindrazana et al. (2011)
*M.fuliginosus*	KIZ20230307	This study	*M.africanus*	MN064734	Lutz et al. (2019)
*M.fuliginosus*	KIZ20230274	This study	*M.griveaudi*	FJ232802	Weyeneth et al. (2008)
*M.fuliginosus*	KIZ20230270	This study	*M.sororculus*	JF440286	Ramasindrazana et al. (2011)
*M.fuliginosus*	MW054886	Ruedi et al. (2021)	*M.petersoni*	EU091258	Goodman et al. (2008)
*M.fuliginosus*	OR468074	Direct Submission	*M.paululus*	MN504402	Demos et al. (2019)
*M.fuliginosus*	AB085737	Sakai et al. (2003)	*M.natalensis*	OP157142	Yi et al. (2024)
*M.pusillus*	KIZ20231111	This study	*M.egeri*	MN504382	Demos et al. (2019)
*M.pusillus*	KIZ20230871	This study	*M.mossambicus*	OQ224761	Benda et al. (2022)
*M.pusillus*	KIZ20231102	This study	*M.brachytragos*	MN504225	Demos et al. (2019)
*M.pusillus*	KIZ20230869	This study	*M.griffithsi*	JF440240	Ramasindrazana et al. (2011)
*M.pusillus*	MN366288	Direct Submission	*M.maghrebensis*	KP455388	Benda et al. (2014)
*M.pusillus*	OR468083	Direct Submission	*M.aelleni*	MN504218	Demos et al. (2019)
*M.pusillus*	MW054888	Ruedi et al. (2021)	*M.ambohitrensis*	MN504221	Demos et al. (2019)
*M.pusillus*	ON640680	Wu et al. (2022)	*K.furva*	MH208502	Direct Submission

**Table 2.** Morphometric data on three species of the genus *Miniopterus*.

Variables	*M.magnater*			*M.fuliginosus*		*M.pusillus*	
	This study n =6	Saikia et al. (2020) n = 12	Kusuminda et al. (2022)	This study n = 6	Kusuminda et al. (2022)	This study n = 6	Kusuminda et al. (2022)
TL	60.70±2.96 (56.24-64.45)	-	59.0±2.00 (56-64 n = 15)	58.64±3.29 (54.78-64.08)	54.2±5.35 (46-60 n = 8)	49.94±3.10 (46.50-54.84)	47.9±2.63 (43-51 n = 7)
FA	50.93±1.38 (48.87-52.30)	-	50.5±1.21 (48-52 n = 16)	48.82±0.84 (47.53-48.34)	46.9±2.39 (42-49 n = 10)	41.26±0.69 (40.43-42.27)	41.7±0.90 (39-42 n = 9)
HB	64.55±3.31 (59.39-66.78)	-	61.0±2.44 (56-65 n = 14)	55.83±1.35 (54.31-57.45)	55.2±4.74 (47-63 n = 10)	50.78±1.96 (49.01-54.26)	48.2±4.64 (42-57 n = 9)
TIB	22.66±0.83 (20.77-23.02)	-	21.2±0.84 (20-22 n = 16)	20.50±0.30 (20.13-20.96)	19.2±1.21 (17-20 n = 9)	17.26±0.62 (16.37-18.06)	17.1±0.78 (15-17 n = 9)
E	10.99±1.38 (9.74-13.04)	-	12.4±1.18 (10.5-14.4 n = 16)	12.07±0.70 (11.45-13.40)	12.3±0.57 (11.7-12.9 n = 4)	9.56±1.02 (8.17-11.11)	10.4±0.43 (10-11 n = 4)
GTL	16.68±0.27 (16.40-17.13)	16.73 (16.5-16.8)	16.7±0.19 (16.4-17.0 n = 13)	15.80±0.33 (15.20-16.19)	15.8±0.26 (15.3-16.4 n = 14)	13.89±0.21 (13.62-14.24)	14.1±0.22 (13.8-14.5 n = 7)
POB	4.17±0.08 (4.06-4.27)	4.29 (4.2-4.4)	4.1±0.11 (4.0-4.3 n = 13)	3.99±0.09 (3.84-4.07)	3.9±0.05 (3.9-4.1 n = 14)	3.65±0.07 (3.58-3.76)	3.6±0.08 (3.5-3.7 n = 7)
MAW	9.24±0.24 (8.88-9.54)	9.33 (9.2-9.5)	9.2±0.24 (8.8-9.5 n = 12)	8.58±0.16 (8.40-8.81)	8.8±0.12 (8.6-9.0 n = 14)	7.77±0.13 (7.61-7.94)	8.0±0.08 (7.9-8.1 n = 7)
M3-M3	7.50±0.13 (7.30-7.60)	7.46 (7.0-7.7)	7.37±0.21 (7.1-7.7 n = 13)	6.80±0.19 (6.59-7.13)	6.71±0.15 (6.4-7.0 n = 14)	5.66±0.11 (5.54-5.81)	5.77±0.07 (5.6-5.8 n = 7)
C-M3	6.67±0.13 (6.45-6.80)	6.85 (6.8-7.1)	6.72±0.10 (6.5-6.9 n = 13)	6.23±0.12 (6.09-6.43)	6.18±0.11 (6.0-6.4 n = 14)	5.22±0.15 (5.03-5.41)	5.33±0.07 (5.2-5.4 n = 7)
C-C	5.26±0.11 (5.13-5.37)	5.23 (5.2-5.3)	5.20±0.19 (4.8-5.5 n = 13)	4.78±0.18 (4.47-4.98)	4.61±0.19 (4.3-4.9 n = 14)	3.99±0.10 (3.86-4.15)	4.13±0.06 (4.0-4.2 n = 7)
ML	12.73±0.34 (12.17-13.19)	12.81 (12.7-12.9)	12.4±0.35 (12.0-13.0 n = 13)	11.92±0.30 (11.41-12.26)	11.4±0.20 (11.1-11.9 n = 14)	10.23±0.17 (10.08-10.52)	9.9±0.12 (9.7-10.0 n = 7)
3mt	43.12±0.50 (42.64-43.93)	-	47.1±0.47 (46-48 n = 8)	42.49±0.50 (41.93-43.09)	42.7±2.38 (38-45 n = 9)	35.56±1.22 (33.66-36.78)	38.1±0.81 (37-39 n = 6)
1ph3mt	12.18±0.49 (12.01-12.76)	-	-	10.99±0.17 (10.66-11.13)	-	9.79±0.32 (9.45-10.39)	-
4mt	40.62±0.81 (39.49-41.46)	-	44.6±0.68 (43-45 n = 8)	40.17±0.77 (39.53-41.59)	41.0±2.41 (36-43 n = 9)	34.53±0.77 (33.47-35.69)	36.7±1.08 (34-37 n = 6)
1ph4mt	10.51±0.28 (10.14-10.91)	-	-	9.80±0.19 (9.45-10.00)	-	8.31±0.22 (7.99-8.62)	-
5mt	36.51±1.62 (34.60-38.74)	-	40.1±0.39 (39-40 n = 8)	36.22±0.74 (35.35-37.38)	37.9±1.82 (34-39 n = 9)	31.71±0.76 (30.92-32.95)	34.5±0.97 (32-35 n = 6)
1ph5mt	10.65±0.32 (10.21-10.99)	-	-	10.04±0.41 (9.34-10.36)	-	8.56±0.39 (7.95-9.13)	-

**Table 3.** Significance test results shown by LSD multiple comparisons results under one-way analysis of variance (ANOVA). * indicates the significance level of 0.05 for the difference between the means. A: *Miniopterusmagnater*; B: *Miniopterusfuliginosus*; C: *Miniopteruspusillus*. P: P-value of the significance test. P＜0.05 was considered to indicate a significant difference, P＜0.01 was considered to indicate an extremely significant difference.

Dependent variable	A and B		A and C		B and C	
	Mean difference	P	Mean difference	P	Mean difference	P
TL	1.31667	0.476	10.02500*	＜0.001	8.70833*	＜0.001
FA	1.77000*	0.008	9.32667*	＜0.001	7.55667*	＜0.001
HB	7.85667*	＜0.001	12.90667*	＜0.001	5.05000*	0.002
TIB	1.84333*	＜0.001	5.09000*	＜0.001	3.24667*	＜0.001
E	-0.73167	0.254	1.77667*	0.012	2.50833*	0.001
GTL	0.89000*	＜0.001	2.79333*	＜0.001	1.90333*	＜0.001
POB	0.17833*	0.001	0.52167*	＜0.001	0.34333*	＜0.001
MAW	0.59500*	＜0.001	1.40500*	＜0.001	0.81000*	＜0.001
M3-M3	0.66500*	＜0.001	1.80833*	＜0.001	1.14333*	＜0.001
C-M3	0.44333*	＜0.001	1.45667*	＜0.001	1.01333*	＜0.001
C-C	0.45167*	＜0.001	1.24500*	＜0.001	0.79333*	＜0.001
ML	0.84667*	＜0.001	2.53833*	＜0.001	1.69167*	＜0.001
3mt	0.55333	0.257	7.47667*	＜0.001	6.92333*	＜0.001
1ph3mt	1.16000*	＜0.001	2.36333*	＜0.001	1.20333*	＜0.001
4mt	0.31000	0.502	5.94667*	＜0.001	5.63667*	＜0.001
1ph4mt	0.68167*	＜0.001	2.17333*	＜0.001	1.49167*	＜0.001
5mt	-0.02833	0.966	4.48667*	＜0.001	4.51500*	＜0.001
1ph5mt	0.58000*	0.018	2.05167*	＜0.001	1.47167*	＜0.001

**Table 4.** Factor loadings and percentage of variance explained for principal component analysis. The extraction method used was principal component analysis and the rotation method was Varimax with Kaiser Normalisation.

Variables	Principal component (PC)		
	1	2	3
TL	0.463	0.828	0.233
FA	0.733	0.494	0.44
HB	0.839	0.466	0.016
TIB	0.766	0.583	0.223
E	0.202	0.189	0.943
GTL	0.846	0.377	0.356
POB	0.766	0.446	0.381
MAW	0.881	0.402	0.187
M3-M3	0.869	0.369	0.303
C-M3	0.844	0.376	0.349
C-C	0.84	0.404	0.309
ML	0.861	0.326	0.35
3mt	0.664	0.533	0.475
1ph3mt	0.87	0.323	0.261
4mt	0.642	0.546	0.489
1ph4mt	0.849	0.378	0.339
5mt	0.495	0.669	0.512
1ph5mt	0.815	0.352	0.349
Eigenvalues	15.849	0.461	0.211
Variance explained (%)	88.048	5.206	2.561
