# Supplementary material for: New record of Miniopterusmagnater (Chiroptera, Miniopteridae) from south-western China and a comparative study of three species of Miniopterus in China
Source: Biodivers Data J. 2024 Sep 13;12:e129879. doi: 10.3897/BDJ.12.e129879 (PMC11415622; doi:10.3897/BDJ.12.e129879)
Supplement: Supplementary material 1 — Uncorrected P-distances (%) amongst sequences. [file bdj-12-e129879-s001.docx]

**Supplementary material 1:** Uncorrected P-distances (%) amongst sequences. 1-7: M. magnater, 1: KIZ20230270, 2: KIZ20230274, 3: KIZ20230307, 4: MW054887, 5: EF517307, 6: ON640726, 7: ON640727. 8-14: M. fuliginosus, 8: KIZ20230869, 9: KIZ20230871, 10: KIZ20231102, 11: KIZ20231111, 12: AB085735, 13: MW054886, 14: OR468074. 15-21: M. pusillus, 15: KIZ20230263, 16: KIZ20230311, 17: KIZ20230314, 18: MN366288, 19: MW054888, 20: ON640680, 21: OR468083.

|  | 1 | 2 | 3 | 4 | 5 | 6 | 7 | 8 | 9 | 10 | 11 | 12 | 13 | 14 | 15 | 16 | 17 | 18 | 19 | 20 | 21 |
| --- | --- | --- | --- | --- | --- | --- | --- | --- | --- | --- | --- | --- | --- | --- | --- | --- | --- | --- | --- | --- | --- |
| 1 |  |  |  |  |  |  |  |  |  |  |  |  |  |  |  |  |  |  |  |  |  |
| 2 | 0.61 |  |  |  |  |  |  |  |  |  |  |  |  |  |  |  |  |  |  |  |  |
| 3 | 0.84 | 1.13 |  |  |  |  |  |  |  |  |  |  |  |  |  |  |  |  |  |  |  |
| 4 | 0.28 | 0.51 | 0.71 |  |  |  |  |  |  |  |  |  |  |  |  |  |  |  |  |  |  |
| 5 | 1.23 | 1.55 | 1.07 | 1.07 |  |  |  |  |  |  |  |  |  |  |  |  |  |  |  |  |  |
| 6 | 0.56 | 0.82 | 0.44 | 0.44 | 0.62 |  |  |  |  |  |  |  |  |  |  |  |  |  |  |  |  |
| 7 | 0.75 | 1.02 | 0.63 | 0.63 | 0.63 | 0.18 |  |  |  |  |  |  |  |  |  |  |  |  |  |  |  |
| 8 | 6.10 | 6.44 | 5.88 | 5.99 | 6.45 | 5.67 | 5.88 |  |  |  |  |  |  |  |  |  |  |  |  |  |  |
| 9 | 6.63 | 7.12 | 6.42 | 6.52 | 6.97 | 6.21 | 6.42 | 1.16 |  |  |  |  |  |  |  |  |  |  |  |  |  |
| 10 | 6.42 | 6.79 | 6.20 | 6.31 | 6.77 | 5.99 | 6.20 | 0.97 | 1.17 |  |  |  |  |  |  |  |  |  |  |  |  |
| 11 | 6.74 | 7.02 | 6.51 | 6.63 | 7.11 | 6.29 | 6.52 | 1.43 | 1.64 | 1.54 |  |  |  |  |  |  |  |  |  |  |  |
| 12 | 6.18 | 6.44 | 6.02 | 6.12 | 6.54 | 5.82 | 6.16 | 0.39 | 1.32 | 0.97 | 1.43 |  |  |  |  |  |  |  |  |  |  |
| 13 | 6.59 | 7.12 | 6.21 | 6.31 | 6.73 | 6.01 | 6.35 | 0.97 | 0.94 | 0.97 | 1.43 | 1.24 |  |  |  |  |  |  |  |  |  |
| 14 | 6.39 | 6.78 | 6.11 | 6.21 | 6.63 | 5.92 | 6.25 | 0.48 | 1.13 | 1.07 | 1.12 | 0.62 | 0.97 |  |  |  |  |  |  |  |  |
| 15 | 13.51 | 13.89 | 13.34 | 13.12 | 13.74 | 13.01 | 13.10 | 14.33 | 13.73 | 14.44 | 13.96 | 13.71 | 13.90 | 13.80 |  |  |  |  |  |  |  |
| 16 | 13.39 | 13.77 | 13.45 | 13.23 | 13.85 | 13.12 | 12.99 | 14.45 | 13.85 | 14.56 | 14.09 | 14.04 | 14.23 | 14.12 | 0.53 |  |  |  |  |  |  |
| 17 | 13.51 | 13.89 | 13.34 | 13.12 | 13.74 | 13.01 | 13.10 | 14.33 | 13.73 | 14.44 | 13.96 | 13.71 | 13.90 | 13.80 | 0.00 | 0.53 |  |  |  |  |  |
| 18 | 13.39 | 13.77 | 13.24 | 13.01 | 13.63 | 12.90 | 12.99 | 14.21 | 13.61 | 14.32 | 13.84 | 13.60 | 13.79 | 13.69 | 0.09 | 0.62 | 0.09 |  |  |  |  |
| 19 | 13.51 | 13.89 | 13.34 | 13.12 | 13.74 | 13.01 | 13.10 | 14.33 | 13.73 | 14.44 | 13.96 | 13.71 | 13.90 | 13.80 | 0.00 | 0.53 | 0.00 | 0.09 |  |  |  |
| 20 | 13.60 | 13.89 | 13.60 | 13.37 | 14.01 | 13.25 | 13.19 | 14.45 | 13.83 | 14.56 | 14.08 | 13.97 | 14.17 | 14.06 | 0.09 | 0.63 | 0.09 | 0.18 | 0.09 |  |  |
| 21 | 13.51 | 13.89 | 13.34 | 13.12 | 13.74 | 13.01 | 13.10 | 14.33 | 13.73 | 14.44 | 13.96 | 13.71 | 13.90 | 13.80 | 0.00 | 0.53 | 0.00 | 0.09 | 0.00 | 0.09 |  |
