# Supplementary material for: New record of Miniopterusmagnater (Chiroptera, Miniopteridae) from south-western China and a comparative study of three species of Miniopterus in China
Source: Biodivers Data J. 2024 Sep 13;12:e129879. doi: 10.3897/BDJ.12.e129879 (PMC11415622; doi:10.3897/BDJ.12.e129879)
Supplement: Supplementary material 2 — LSD multiple comparisons [file bdj-12-e129879-s002.docx]

Supporting material 2: Results of LSD multiple comparisons of morphometric data of three specimens of species of the genus *Miniopterus* collected in this study. Among them, Group 1 is the specimens of *Miniopterus magnater*, Group 2 is the specimens of *Miniopterus fuliginosus*, and Group 3 is the specimens of *Miniopterus pusillus*.

| LSD Multiple Comparison | | | | | | | |
| --- | --- | --- | --- | --- | --- | --- | --- |
| implicit variable | (I) Groups | (J) Group | Difference in mean values (I-J) | standard error | significance | 95% confidence interval(math.) | |
|  |  |  |  |  |  | lower limit | limit |
| TL | 1 | 2 | 1.31667 | 1.80074 | 0.476 | -2.5215 | 5.1548 |
|  |  | 3 | 10.02500* | 1.80074 | <0.01 | 6.1868 | 13.8632 |
|  | 2 | 1 | -1.31667 | 1.80074 | 0.476 | -5.1548 | 2.5215 |
|  |  | 3 | 8.70833* | 1.80074 | <0.01 | 4.8702 | 12.5465 |
|  | 3 | 1 | -10.02500* | 1.80074 | <0.01 | -13.8632 | -6.1868 |
|  |  | 2 | -8.70833* | 1.80074 | <0.01 | -12.5465 | -4.8702 |
| FA | 1 | 2 | 1.77000* | 0.58352 | 0.008 | 0.5263 | 3.0137 |
|  |  | 3 | 9.32667* | 0.58352 | <0.01 | 8.0829 | 10.5704 |
|  | 2 | 1 | -1.77000* | 0.58352 | 0.008 | -3.0137 | -0.5263 |
|  |  | 3 | 7.55667* | 0.58352 | <0.01 | 6.3129 | 8.8004 |
|  | 3 | 1 | -9.32667* | 0.58352 | <0.01 | -10.5704 | -8.0829 |
|  |  | 2 | -7.55667* | 0.58352 | <0.01 | -8.8004 | -6.3129 |
| HB | 1 | 2 | 7.85667* | 1.35883 | <0.01 | 4.9604 | 10.7529 |
|  |  | 3 | 12.90667* | 1.35883 | <0.01 | 10.0104 | 15.8029 |
|  | 2 | 1 | -7.85667* | 1.35883 | <0.01 | -10.7529 | -4.9604 |
|  |  | 3 | 5.05000* | 1.35883 | 0.002 | 2.1537 | 7.9463 |
|  | 3 | 1 | -12.90667* | 1.35883 | <0.01 | -15.8029 | -10.0104 |
|  |  | 2 | -5.05000* | 1.35883 | 0.002 | -7.9463 | -2.1537 |
| TIB | 1 | 2 | 1.84333* | 0.35935 | <0.01 | 1.0774 | 2.6093 |
|  |  | 3 | 5.09000* | 0.35935 | <0.01 | 4.3241 | 5.8559 |
|  | 2 | 1 | -1.84333* | 0.35935 | <0.01 | -2.6093 | -1.0774 |
|  |  | 3 | 3.24667* | 0.35935 | <0.01 | 2.4807 | 4.0126 |
|  | 3 | 1 | -5.09000* | 0.35935 | <0.01 | -5.8559 | -4.3241 |
|  |  | 2 | -3.24667* | 0.35935 | <0.01 | -4.0126 | -2.4807 |
| E | 1 | 2 | -0.73167 | 0.6175 | 0.254 | -2.0478 | 0.5845 |
|  |  | 3 | 1.77667* | 0.6175 | 0.012 | 0.4605 | 3.0928 |
|  | 2 | 1 | 0.73167 | 0.6175 | 0.254 | -0.5845 | 2.0478 |
|  |  | 3 | 2.50833* | 0.6175 | 0.001 | 1.1922 | 3.8245 |
|  | 3 | 1 | -1.77667* | 0.6175 | 0.012 | -3.0928 | -0.4605 |
|  |  | 2 | -2.50833* | 0.6175 | 0.001 | -3.8245 | -1.1922 |
| GTL | 1 | 2 | .89000* | 0.15886 | <0.01 | 0.5514 | 1.2286 |
|  |  | 3 | 2.79333* | 0.15886 | <0.01 | 2.4547 | 3.1319 |
|  | 2 | 1 | -.89000* | 0.15886 | <0.01 | -1.2286 | -0.5514 |
|  |  | 3 | 1.90333* | 0.15886 | <0.01 | 1.5647 | 2.2419 |
|  | 3 | 1 | -2.79333* | 0.15886 | <0.01 | -3.1319 | -2.4547 |
|  |  | 2 | -1.90333* | 0.15886 | <0.01 | -2.2419 | -1.5647 |
| POB | 1 | 2 | .17833* | 0.04561 | 0.001 | 0.0811 | 0.2756 |
|  |  | 3 | .52167* | 0.04561 | <0.01 | 0.4244 | 0.6189 |
|  | 2 | 1 | -.17833* | 0.04561 | 0.001 | -0.2756 | -0.0811 |
|  |  | 3 | .34333* | 0.04561 | <0.01 | 0.2461 | 0.4406 |
|  | 3 | 1 | -.52167* | 0.04561 | <0.01 | -0.6189 | -0.4244 |
|  |  | 2 | -.34333* | 0.04561 | <0.01 | -0.4406 | -0.2461 |
| MAW | 1 | 2 | .59500* | 0.10481 | <0.01 | 0.3716 | 0.8184 |
|  |  | 3 | 1.40500* | 0.10481 | <0.01 | 1.1816 | 1.6284 |
|  | 2 | 1 | -.59500* | 0.10481 | <0.01 | -0.8184 | -0.3716 |
|  |  | 3 | .81000* | 0.10481 | <0.01 | 0.5866 | 1.0334 |
|  | 3 | 1 | -1.40500* | 0.10481 | <0.01 | -1.6284 | -1.1816 |
|  |  | 2 | -.81000* | 0.10481 | <0.01 | -1.0334 | -0.5866 |
| M3-M3 | 1 | 2 | .66500* | 0.08438 | <0.01 | 0.4851 | 0.8449 |
|  |  | 3 | 1.80833* | 0.08438 | <0.01 | 1.6285 | 1.9882 |
|  | 2 | 1 | -.66500* | 0.08438 | <0.01 | -0.8449 | -0.4851 |
|  |  | 3 | 1.14333* | 0.08438 | <0.01 | 0.9635 | 1.3232 |
|  | 3 | 1 | -1.80833* | 0.08438 | <0.01 | -1.9882 | -1.6285 |
|  |  | 2 | -1.14333* | 0.08438 | <0.01 | -1.3232 | -0.9635 |
| C-M3 | 1 | 2 | .44333* | 0.07699 | <0.01 | 0.2792 | 0.6074 |
|  |  | 3 | 1.45667* | 0.07699 | <0.01 | 1.2926 | 1.6208 |
|  | 2 | 1 | -.44333* | 0.07699 | <0.01 | -0.6074 | -0.2792 |
|  |  | 3 | 1.01333* | 0.07699 | <0.01 | 0.8492 | 1.1774 |
|  | 3 | 1 | -1.45667* | 0.07699 | <0.01 | -1.6208 | -1.2926 |
|  |  | 2 | -1.01333* | 0.07699 | <0.01 | -1.1774 | -0.8492 |
| C-C | 1 | 2 | .45167* | 0.07945 | <0.01 | 0.2823 | 0.621 |
|  |  | 3 | 1.24500* | 0.07945 | <0.01 | 1.0756 | 1.4144 |
|  | 2 | 1 | -.45167* | 0.07945 | <0.01 | -0.621 | -0.2823 |
|  |  | 3 | .79333* | 0.07945 | <0.01 | 0.624 | 0.9627 |
|  | 3 | 1 | -1.24500* | 0.07945 | <0.01 | -1.4144 | -1.0756 |
|  |  | 2 | -.79333* | 0.07945 | <0.01 | -0.9627 | -0.624 |
| ML | 1 | 2 | .84667* | 0.16218 | <0.01 | 0.501 | 1.1923 |
|  |  | 3 | 2.53833* | 0.16218 | <0.01 | 2.1927 | 2.884 |
|  | 2 | 1 | -.84667* | 0.16218 | <0.01 | -1.1923 | -0.501 |
|  |  | 3 | 1.69167* | 0.16218 | <0.01 | 1.346 | 2.0373 |
|  | 3 | 1 | -2.53833* | 0.16218 | <0.01 | -2.884 | -2.1927 |
|  |  | 2 | -1.69167* | 0.16218 | <0.01 | -2.0373 | -1.346 |
| 3mt | 1 | 2 | 0.55333 | 0.46979 | 0.257 | -0.448 | 1.5547 |
|  |  | 3 | 7.47667* | 0.46979 | <0.01 | 6.4753 | 8.478 |
|  | 2 | 1 | -0.55333 | 0.46979 | 0.257 | -1.5547 | 0.448 |
|  |  | 3 | 6.92333* | 0.46979 | <0.01 | 5.922 | 7.9247 |
|  | 3 | 1 | -7.47667* | 0.46979 | <0.01 | -8.478 | -6.4753 |
|  |  | 2 | -6.92333* | 0.46979 | <0.01 | -7.9247 | -5.922 |
| 1ph3mt | 1 | 2 | 1.16000* | 0.20246 | <0.01 | 0.7285 | 1.5915 |
|  |  | 3 | 2.36333* | 0.20246 | <0.01 | 1.9318 | 2.7949 |
|  | 2 | 1 | -1.16000* | 0.20246 | <0.01 | -1.5915 | -0.7285 |
|  |  | 3 | 1.20333* | 0.20246 | <0.01 | 0.7718 | 1.6349 |
|  | 3 | 1 | -2.36333* | 0.20246 | <0.01 | -2.7949 | -1.9318 |
|  |  | 2 | -1.20333* | 0.20246 | <0.01 | -1.6349 | -0.7718 |
| 4mt | 1 | 2 | 0.31 | 0.45101 | 0.502 | -0.6513 | 1.2713 |
|  |  | 3 | 5.94667* | 0.45101 | <0.01 | 4.9854 | 6.908 |
|  | 2 | 1 | -0.31 | 0.45101 | 0.502 | -1.2713 | 0.6513 |
|  |  | 3 | 5.63667* | 0.45101 | <0.01 | 4.6754 | 6.598 |
|  | 3 | 1 | -5.94667* | 0.45101 | <0.01 | -6.908 | -4.9854 |
|  |  | 2 | -5.63667* | 0.45101 | <0.01 | -6.598 | -4.6754 |
| 1ph4mt | 1 | 2 | .68167* | 0.13472 | <0.01 | 0.3945 | 0.9688 |
|  |  | 3 | 2.17333* | 0.13472 | <0.01 | 1.8862 | 2.4605 |
|  | 2 | 1 | -.68167* | 0.13472 | <0.01 | -0.9688 | -0.3945 |
|  |  | 3 | 1.49167* | 0.13472 | <0.01 | 1.2045 | 1.7788 |
|  | 3 | 1 | -2.17333* | 0.13472 | <0.01 | -2.4605 | -1.8862 |
|  |  | 2 | -1.49167* | 0.13472 | <0.01 | -1.7788 | -1.2045 |
| 5mt | 1 | 2 | -0.02833 | 0.64468 | 0.966 | -1.4024 | 1.3458 |
|  |  | 3 | 4.48667* | 0.64468 | <0.01 | 3.1126 | 5.8608 |
|  | 2 | 1 | 0.02833 | 0.64468 | 0.966 | -1.3458 | 1.4024 |
|  |  | 3 | 4.51500* | 0.64468 | <0.01 | 3.1409 | 5.8891 |
|  | 3 | 1 | -4.48667* | 0.64468 | <0.01 | -5.8608 | -3.1126 |
|  |  | 2 | -4.51500* | 0.64468 | <0.01 | -5.8891 | -3.1409 |
| 1ph5mt | 1 | 2 | .58000* | 0.21739 | 0.018 | 0.1166 | 1.0434 |
|  |  | 3 | 2.05167* | 0.21739 | <0.01 | 1.5883 | 2.515 |
|  | 2 | 1 | -.58000* | 0.21739 | 0.018 | -1.0434 | -0.1166 |
|  |  | 3 | 1.47167* | 0.21739 | <0.01 | 1.0083 | 1.935 |
|  | 3 | 1 | -2.05167* | 0.21739 | <0.01 | -2.515 | -1.5883 |
|  |  | 2 | -1.47167* | 0.21739 | <0.01 | -1.935 | -1.0083 |
